# Dietary Patterns and Gallstone Risks in Chinese Adults: A Cross-sectional Analysis of the China Multi-Ethnic Cohort Study

**DOI:** 10.2188/jea.JE20220039

**Published:** 2023-09-05

**Authors:** Chan Nie, Tingting Yang, Ziyun Wang, Deji Suolang, Songmei Wang, Kangzhuo Baima, Li Wei, Hua Ling, Leilei Liu, Qibing Zeng, Zixiu Qin, Haojiang Zuo, Feng Hong

**Affiliations:** 1 School of Public Health, the Key Laboratory of Environmental Pollution Monitoring and Disease Control, Ministry of Education, Guizhou Medical University, Guiyang, China; 2 Tibet Center for Disease Control and Prevention, Lhasa, China; 3 School of Public Health, Kunming Medical University, Kunming, China; 4 Division of Pulmonary Diseases, State Key Laboratory of Biotherapy, West China Hospital of Sichuan University, Chengdu, China; 5 School of Medicine, Tibet University, Lhasa, China; 6 Wuhou District Center for Disease Control and Prevention, Chengdu, China; 7 Chongqing Center for Disease Control and Prevention, Chongqing, China; 8 West China School of Public Health and West China Fourth Hospital, Sichuan University, Chengdu, China; 9 Food Safety Monitoring and Risk Assessment Key Laboratory of Sichuan Province, Chengdu, China

**Keywords:** dietary pattern, gallstone disease, Chinese, epidemiological study

## Abstract

**Background:**

Little is known about the association between a plant-based diet and the risk of gallstone disease (GD), especially in developing counties. We tested the hypothesis that shifting dietary patterns would be related to the risk of GD, and that the Mediterranean diet (MED) adjusted for China would be beneficial for lowering risk of GD.

**Methods:**

Data were extracted from the baseline survey of the China Multi-Ethnic Cohort study. An alternative Mediterranean diet (aMED) score was assessed based on a food frequency questionnaire, and three posteriori dietary patterns (the modern dietary pattern, the coarse grain dietary pattern, and the rice dietary pattern) were identified using factor analysis. Multivariable logistic regression models were developed to evaluate the association between dietary patterns and GD risks.

**Results:**

A total of 89,544 participants were included. The prevalence of GD was 7.5%. Comparing the highest with lowest quintiles, aMED was associated with an increased risk of GD (OR 1.13; 95% CI, 1.04–1.24; *P*_trend_ = 0.003), whereas the rice dietary pattern was inversely related to GD risk (OR 0.79; 95% CI, 0.71–0.87; *P*_trend_ < 0.001). In stratified analysis, the rice dietary pattern had a stronger inverse association in the subgroups of females, older, urban, and overweight participants, and those with diabetes—factors associated with higher rates of GD in previous studies.

**Conclusion:**

Higher adherence to the rice dietary pattern was associated with a lower risk of GD. For high-risk populations, making some shift to a traditional agricultural diet might help with primary prevention of GD.

## INTRODUCTION

Gallstone disease (GD) is a common gastrointestinal disease. It is highly prevalent in western countries, with an average prevalence of 10–20% among adults.^1^^–^^3^ GD has been reported to increase the risks of major chronic diseases, such as type 2 diabetes, cardiovascular diseases, and gallbladder cancer.^4^^–^^6^ Evidence has suggested that dietary intervention plays a vital role in GD primary prevention.^7^ Traditional western diet, characterized by high intake of refined grains, fat, and sweets, has been associated with increased risk of GD.^8^^,^^9^ On the contrary, a Mediterranean dietary pattern (MED), rich in vegetables, fruits, whole-meal cereals, nuts, olive oil, and fish, was reported to be associated with a lower risk of GD.^10^^,^^11^ Unfortunately, these studies are from western countries, and data on the association between diet and GD in Asian countries is limited. Moreover, some studies were limited to specific food components or isolated nutrients, which might neglect possible interactions of food.^12^^–^^14^ As a traditional rice-based country, China’s dietary pattern is significantly different from that in the west. There was a cohort study, conducted in Taiwan, that investigated the association between diet and GD, but the target population was vegetarians who completely avoided meat or fish.^15^ According to previous studies, the effects of a vegetarian diet on the risk of GD remain controversial.^16^^,^^17^

In China, the rate of GD averages between 5% and 11% and is expected to increase in the future.^18^^–^^20^ To the best of our knowledge, there are no existing studies on the association between dietary patterns and GD risks in mainland China. We hypothesized that shifting dietary patterns would be related to the risk of GD, and the MED diet pattern adjusted for China would be beneficial for lowering risk of GD.

This study was based on the baseline survey of the China Multi-Ethnic Cohort (CMEC) study, which is a large-scale epidemiological study conducted in Southwest China, covering the Sichuan Basin, Qinghai-Tibet Plateau, and Yunnan-Guizhou Plateau, with great diversity in economic status, ethnicity, and diet habit. We developed the Mediterranean diet score adapted to the Southwest China diet (ie, the alternative Mediterranean diet [aMED] score) and the posteriori dietary patterns to investigate the effects of the a priori aMED dietary pattern and posteriori dietary patterns on development of GD in Chinese adults.

## METHODS

### Study population

This cross-sectional analysis used baseline data from the CMEC study. Detailed information about the CMEC study has been previously reported.^21^ Briefly, a total of 99,556 middle-aged adults were enrolled in our cohort from May 2018 to September 2019. Participants were from seven ethnic groups, including Tibetan, Yi, Miao, Bai, Bouyei, Dong, and Han, from five provinces (Sichuan, Chongqing, Yunnan, Guizhou, and Tibet) of Southwest China. The baseline survey consisted of an electronic questionnaire with face-to-face interviews, medical examinations, and clinical laboratory tests.

In this study, participants with a history of cholecystectomy or without abdominal ultrasound examination were excluded. In addition, self-reported cases of GD were also excluded. Finally, 89,544 adults (35,702 men and 53,842 women) aged 30–79 years with complete and plausible diet-related data were available for the present analysis (eFigure 1). Ethical approval was received from the Sichuan University Medical Ethical Review Board. All the participants signed the informed consent.

### Dietary assessment

Habitual dietary intake during the preceding 12 months was assessed using a food frequency questionnaire (FFQ) in the baseline survey. The FFQ comprises a list of 13 commonly consumed foods in southwest China: tubers, red and processed meats, poultry, fish/seafood, eggs, fresh vegetables, soybean products, preserved vegetables, fresh fruits, dairy products, rice, wheat products, coarse grain (including corn, sorghum, and groundnut). The food consumption of each group was reported by participants, including the quantity (how many grams per meal according to a standard serving size molds) and frequency (how often timed per day, per week, per month, or per year did they consume during the past 12 months). The consumption of alcohol, tea, beverages, cooking oil, and salt was also recorded in separate sections. In the case of oil and salt, having enquired the household consumption and the number of people eating each meal during the past month, we calculated the daily consumption of cooking oil and salt of each person. The oil types of consumption were also collected, including vegetable oil/sesame oil, peanut oil, soybean oil, lard, and blended oil. The FFQ’s reproducibility and validity have been reported previously, except for the validity of oil and salt, in 24-hour dietary recall.^22^

### Dietary patterns

We developed the aMED score, which was modified from the traditional MED score and adapted to the Southwest China diet.^22^^,^^23^ The aMED score focuses on eight kinds of intakes (vegetables, legumes, fruit, whole grains, fish, mono-unsaturated fatty acids [MUFA]:saturated fatty acids [SFA] ratio, red and processed meats, and ethanol). We modified the original scale for this study by excluding the dairy group, including only red and processed meats for the meat group, and excluding nuts due to no available information. A score of 1 to 5 was assigned for each kind of food according to the quintile of average food intake, and ethanol was scored according to the moderate alcohol intake criteria. The aMED score was calculated as the sum of points and was expressed in a range from 8 to 40. More details about the scoring criteria can be seen in eTable 1.

To generate posteriori dietary patterns, the consumption of eighteen food groups intake variables were included in the principal component factor analysis, and varimax (orthogonal) rotation was performed. To identify the number of dietary patterns to retain, the eigenvalues (from the correlation matrix of the standardized variables) >1.4 of a criterion, which was the break-point identified in the scree plot of the eigenvalues, and the characteristics of the ethnic regions were considered. As our previous study has found,^22^ three dietary patterns (the modern dietary pattern, the coarse grain dietary pattern, and the rice dietary pattern) could obtain the best discrimination among different ethnic regions, which were highly related to three geographic regions: the Sichuan Basin, the Qinghai-Tibet Plateau, and the Yunnan-Guizhou Plateau. The three dietary patterns explained 28.0% of the total variance. The modern dietary pattern was characterized by high loadings of fish/seafood, poultry, eggs, fresh fruits, fresh vegetables, and dairy products, predominated by the Han majority population in two more developed cities (Chongqing and Chengdu) in the Sichuan Basin. The coarse grain dietary pattern comprised higher consumption of coarse grain, wheat products, tubers, tea, soybean products, preserved vegetables, and red and processed meats, and was lacking fresh vegetables, features of the high-altitude Tibetan dietary pattern. The rice dietary pattern included high loadings of animal oil, rice, preserved vegetables, alcohol, salt, soybean products, and fresh vegetables, suggesting a traditional agricultural dietary pattern followed by various ethnic minority populations in the Yunnan-Guizhou Plateau (Table 1).

**Table 1.** Factor loading matrix for three dietary patterns from Principal Component Factor Analysis

	Modern	Coarse grain	Rice
Rice	0.08	−0.02	**0.52**
Soybean products	0.27	**0.24**	**0.25**
Tubers	0.16	**0.54**	0.16
Wheat products	−0.05	**0.63**	−0.03
Coarse grain	−0.01	**0.63**	−0.31
Preserved vegetables	0.13	**0.21**	**0.35**
Fresh vegetables	**0.44**	−0.10	**0.17**
Fresh fruits	**0.45**	0.02	−0.13
Poultry	**0.60**	0.06	0.10
Fish/sea food	**0.63**	−0.04	0.11
Red and processed meats	0.25	**0.13**	0.12
Eggs	**0.49**	0.09	−0.14
Dairy products	**0.42**	0.07	−0.50
Vegetable oil	**0.31**	−0.13	−0.24
Animal oil	−0.18	−0.11	**0.63**
Salt	−0.01	−0.21	**0.28**
Alcohol	0.17	0.09	**0.28**
Tea	−0.02	**0.51**	0.03

Bolded values are marked for the top seven loadings of foods in each dietary patterns.

The three dietary patterns explained 28.0% of the total variance.

### Definition of GD

GD was determined by a B-ultrasound examination (SIUI Apogee1200; Shantou Institute of Ultrasonic Instruments Co., Shantou, China). The presence of echoes within the gallbladder dependent on gravity or attenuation of ultrasonic transmission (acoustic shadowing) was diagnosed as gallstones.^24^ All the doctors received standardized training before the investigation. In our study, biliary sludge was classified into the GD group. Cholecystectomy was defined as the absence of the gallbladder on abdominal ultrasonography. To decrease the misdiagnosis rate of ultrasonography, participants’ surgical history was also considered.

### Statistical analysis

Baseline descriptions were expressed as means and standard deviations for continuous variables and percentages for categorical variables according to quintiles of dietary pattern scores. Multivariable logistic regression models were developed to evaluate the odds ratios (ORs) and 95% confidence intervals (CIs) for GD according to quartiles in different dietary patterns, and the lowest quartile was used as the reference group. Based on the previous literature, we included the following predefined confounders in the multivariable-adjusted models: age, sex, ethnicity, urbanicity, annual family income, occupation, education level, marital status, total energy intake, physical activity, body mass index (BMI), smoking status, regular intake of beverages, regular intake of dietary supplements, menopause status (women only), and history of diabetes. We used two models: one only adjusted for age, sex, and ethnicity, and one fully adjusted using the aforementioned covariates. The linear trend analysis across quartiles in the logistic models was performed by assigning a median value to each quartile of the dietary score and modeling it as a continuous variable.

To facilitate interpretation of the results, we carried out a single-group analysis to evaluate the association of each of the food components with GD risks. All components of food in the FFQ and the aMED score were simultaneously introduced in models, allowing assessment of the relative impact of each component. Furthermore, stratified analysis was also performed to evaluate associations of dietary patterns with GD risk across subgroups of sex, age, urbanicity, BMI, physical activity, history of diabetes, and ethnic group.

To test the sensitivity of our findings, analysis was repeated after excluding those with biliary sludge (*n* = 962). All analyses were performed by SPSS software version 24.0 for Windows (SPSS Inc., Chicago, IL, USA). A two-sided *P* value below 0.05 was deemed statistically significant.

## RESULTS

Basic characteristics are presented by quintiles of different dietary scores in Table 2. Among the 89,544 participants included in this study, the mean age was 51.6 (standard deviation, 11.5) years, 60.1% of the participants were women, 65.5% were rural residents, and 41.7% were from ethnic minority populations. Participants with a higher score of aMED dietary pattern and the modern dietary pattern shared similar characteristics. They were more likely to be younger, urban residents, have a higher education level, higher family income, lower physical activity, and were more likely to report a history of diabetes. On the contrary, compared to participants with a lower dietary score in the coarse grain dietary pattern and the rice dietary pattern, those in higher score quintiles tended to be older, rural residents, have a lower education level, lower family income, and were less likely to report a history of diabetes. Additionally, participants with higher dietary scores of the rice dietary pattern had a lower BMI than those in the lower score group.

**Table 2.** Basic characteristics of study participants across quartiles of various dietary pattern scores

Characteristics	*n*	Overall	aMED		Modern		Coarse grain		Rice	
			
			Q1	Q5	Q1	Q5	Q1	Q5	Q1	Q5
**Dietary score (SD)**	89,544	—	18.1 (1.9)	30.7 (1.7)	−1.2 (0.3)	1.5 (0.7)	−1.0 (0.2)	1.6 (0.9)	−1.3 (0.4)	1.5 (0.6)
**Age, years (SD)**	89,544	51.6 (11.5)	53.3 (11.8)	50.7 (11.1)	53.4 (11.5)	50.0 (11.3)	51.7 (11.3)	52.1 (11.4)	50.3 (12.2)	53.0 (10.5)
**Female sex, %**	53,842	60.1	57.3	61.5	67.4	50.1	70.5	49.4	71.7	46.9
**Annual family income, yuan, %**										
<12,000	15,384	17.2	27.1	8.8	28.2	9.1	22.9	16.2	10.6	23.9
12,000–19,999	16,358	18.3	23.1	13.5	24.4	12.4	19.2	20.7	15.5	21.1
20,000–59,999	32,532	36.4	33.8	36.8	34.5	35.1	34.4	37.5	34.3	38.6
60,000–99,999	13,194	14.8	9.2	20.3	8.1	20.9	12.9	13.2	19.4	9.5
≥100,000	11,973	13.4	6.8	20.7	4.8	22.5	10.7	12.4	20.2	6.8
**Occupation, %**										
Primary industry practitioner	30,923	34.6	47.9	20.7	49.3	17.6	38.6	35.2	16.4	57.0
Secondary industry practitioner	6,480	7.2	6.2	7.6	4.4	9.6	7.7	5.3	5.5	7.6
Tertiary industry practitioner	33,293	37.2	29.4	43.1	32.5	43.6	37.2	35.7	47.6	22.9
Unemployed or other	18,779	21.0	16.5	28.6	13.8	29.3	16.5	23.8	30.5	12.5
**Education level, %**										
No formal school	23,702	26.5	43.0	11.3	47.5	10.4	31.6	32.7	23.7	28.9
Primary school	22,804	25.5	27.9	20.6	29.4	19.5	25.1	27.9	17.3	35.3
Middle and high school	33,235	37.1	23.6	52.0	20.0	51.8	34.0	31.1	39.5	32.4
College or university	9,802	10.9	5.6	16.1	3.1	18.3	9.3	8.3	19.5	3.4
**Married or cohabiting, %**	79,609	88.9	87.2	90.3	86.4	89.7	86.6	90.4	87.1	89.7
**Rural residence, %**	58,614	65.5	80.5	46.7	86.0	45.2	69.6	70.6	46.6	84.0
**Menopausal status in women, %**										
Pre-menopause	24,394	45.3	39.1	49.9	37.7	53.8	43.6	46.1	51.4	41.0
Peri-menopause	3,688	6.8	6.5	7.7	6.6	6.9	7.4	6.2	6.4	6.9
Post-menopause	25,760	47.8	54.4	42.4	55.7	39.3	49.0	47.7	42.2	52.1
**BMI, kg/m^2^, %**										
<24	44,555	49.8	49.1	49.4	49.7	47.7	52.4	43.3	49.0	54.7
24–<28	33,092	37.0	36.0	38.0	36.0	39.0	35.5	40.5	37.3	34.5
≥28	11,747	13.1	14.9	12.6	14.3	13.2	12.2	16.2	13.7	10.9
**Physical activity, METs/day (SD)**	89,172	26.2 (18.3)	27.3 (19.6)	24.5 (16.5)	27.1 (19.8)	24.3 (16.4)	27.3 (18.6)	25.1 (18.4)	20.2 (14.8)	33.4 (20.3)
**Regular smoking, %**										
Never	67,059	74.9	75.0	74.8	81.4	67.3	82.2	67.5	84.9	62.2
Smoking or quit smoking	22,485	25.1	25.0	25.2	18.6	32.7	17.8	32.5	15.1	37.8
**Total energy intake, kcal/day (SD)**	89,544	1,827.9 (617.9)	1,750.4 (653.8)	1,982.32 (569.92)	1,428.2 (503.3)	2,261.5 (614.7)	1,616.2 (568.8)	2,212.6 (641.3)	1,716.5 (577.1)	2,211.3 (622.2)
**Regular beverage intake, %**	6,574	7.3	11.4	4.1	10.5	6.2	3.5	16.3	11.2	4.9
**Dietary supplement, %**	14,460	16.1	9.9	23.2	10.6	20.6	14.6	14.5	20.8	13.3
**Ethnic group, %**										
Sichuan Basin	42,269	47.2	29.8	69.5	17.1	76.9	44.3	34.4	65.0	29.1
Yunnan-Guizhou Plateau	38,030	42.5	50.8	29.1	56.9	20.4	54.6	30.6	9.7	69.1
Qinghai-Tibet Plateau	9,245	10.3	19.4	1.4	26.1	2.7	1.2	35.1	25.4	1.8
**History of diabetes, %**	4,127	4.6	4.5	5.2	4.0	5.2	4.7	4.4	5.9	3.3

aMED, alternative Mediterranean diet; BMI, body mass index; MET, metabolic equivalent of task; Q, quartile; SD, standard deviation.

Data are presented as mean values and standard deviations for continuous variables or percentages (%) for categorical variables.

Associations between GD risks and dietary patterns are presented in Table 3. Overall, 7.5% (6,727) of the participants were newly diagnosed with GD. In model 1 (adjusted for sex, age, and ethnicity), the aMED dietary pattern and the coarse grain dietary pattern were positively associated with GD risk, whereas the rice dietary pattern showed a strong inverse association with GD risk. In the fully adjusted model (adjusted model 2), the associations were attenuated but still significant. Comparing the highest with lowest quintiles, the odds ratios of GD risk were 1.13 (95% CI, 1.04–1.24; *P*_trend_ = 0.003), 1.13 (95% CI, 1.03–1.24; *P*_trend_ = 0.008) and 0.79 (95% CI, 0.71–0.87; *P*_trend_ < 0.001) for aMED, the coarse grain dietary pattern, and the rice dietary pattern, respectively. There was no significant association between the modern dietary pattern and GD risk in model 1, but a marginally inverse association in model 2 with an odd radio of 0.88 (95% CI, 0.80–0.98; *P*_trend_ = 0.064) comparing the highest with lowest quintiles. Interestingly, in the single-food component analysis, we found that MUFA:SFA contributed most to the risk of GD according to aMED, while fresh vegetable and animal oil were beneficial for decreased GD risks (eTable 2 and eTable 3).

**Table 3.** Associations between various dietary patterns and gallstone risks according to quintiles of dietary pattern scores

	Q1	Q2	Q3	Q4	Q5	*P* for trend
Model 1^a^						
aMED	Ref.	1.13 (1.04–1.21)	1.14 (1.05–1.24)	1.27 (1.17–1.37)	1.33 (1.22–1.45)	<0.001
Modern	Ref.	0.95 (0.88–1.03)	1.01 (0.93–1.09)	1.06 (0.97–1.15)	1.05 (0.96–1.15)	0.052
Coarse grain	Ref.	1.06 (0.98–1.15)	1.03 (0.95–1.12)	1.09 (1.00–1.18)	1.13 (1.03–1.24)	0.010
Rice	Ref.	0.92 (0.85–0.99)	0.84 (0.77–0.91)	0.76 (0.70–0.83)	0.61 (0.56–0.67)	<0.001
Model 2^b^						
aMED	Ref.	1.07 (0.99–1.16)	1.05 (0.96–1.14)	1.13 (1.04–1.23)	1.13 (1.04–1.24)	0.003
Modern	Ref.	0.92 (0.84–0.99)	0.93 (0.85–1.01)	0.93 (0.85–1.02)	0.88 (0.80–0.98)	0.064
Coarse grain	Ref.	1.03 (0.95–1.11)	1.00 (0.92–1.09)	1.06 (0.98–1.16)	1.13 (1.03–1.24)	0.008
Rice	Ref.	0.96 (0.89–1.04)	0.92 (0.85–1.00)	0.89 (0.82–0.97)	0.79 (0.71–0.87)	<0.001

aMED, alternative Mediterranean diet; Q, quartile.

Data are presented as odds ratio (95% confidence interval).

^a^Model 1 adjusted for sex, age, and ethnicity.

^b^Model 2 adjusted for model 1 + urbanicity, marital status, education level, annual family income, occupation, physical activity, smoking status, regular intake of beverage, regular intake of dietary supplements, total energy intake, body mass index, and history of diabetes.

**Figure 1.**
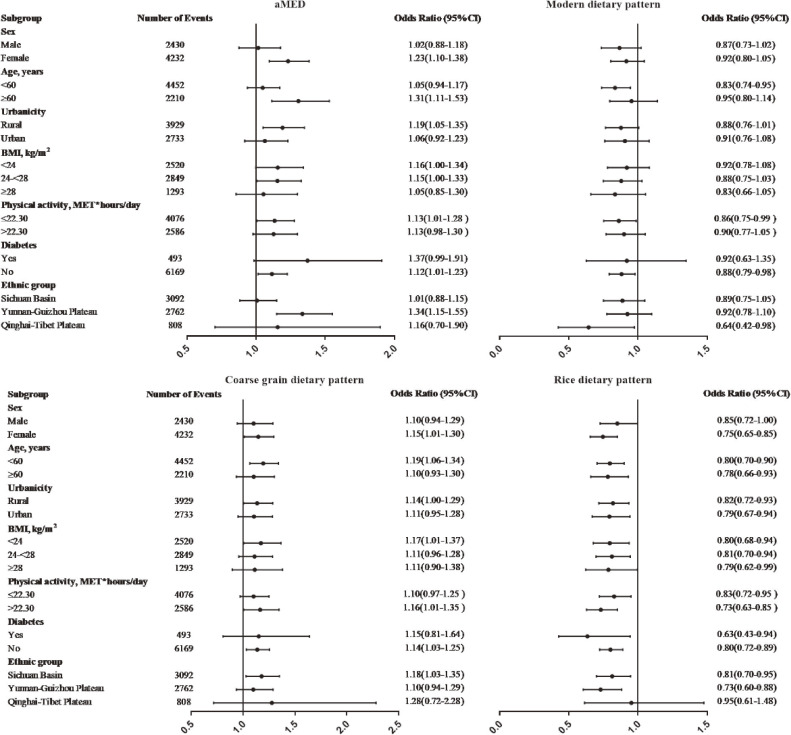
Stratified analysis of estimated associations between various dietary patterns and gallstone risks according to predefined characteristics, by comparing the highest with the lowest quintiles. Analyses were adjusted for sex, age, ethnicity, urbanicity, marital status, education level, annual family income, occupation, physical activity, smoking status, regular intake of beverage, regular intake of dietary supplements, total energy intake, BMI, and history of diabetes, with the exclusion of the stratified variable as appropriate. For the female group, the menopause status was also adjusted. Dots and bars represent adjusted odds ratios and 95% CI, respectively. BMI, body mass index; CI, confidence interval; MET, metabolic equivalent of task.

In the stratified analysis, associations generally did not differ between dietary patterns and GD risks, although not all subgroups indicated a significant difference. It is worth noting that none of the associations were not evident among men. Actually, the prevalence of GD was higher in women than in men (7.9% vs 6.8%) in this study. The aMED dietary pattern showed a positive association with GD risk in females, those 60 years old and above, those without diabetes, and participants in the Yunnan-Guizhou plateau group. For the rice dietary pattern, there were still negative associations with GD risks in all subgroups except the Qinghai-Tibet plateau. Importantly, inverse associations appeared to be stronger among participants who were female, older (≥60 years), urban, overweight (BMI ≥28 kg/m^2^), more physically active (greater than the sample median of 22.30 MET-hours/day), and had a history of diabetes, most of whom are of higher risk of GD.^25^^–^^27^ The modern dietary pattern was inversely related to GD risks only when participants were under 60 years old and free of diabetes (Figure 1).

In sensitivity analysis, after further excluding subjects with biliary sludge in the fully adjusted model, both the associations and linear trend between GD and dietary patterns remained (eFigure 2).

## DISCUSSION

Based on large-scale epidemiological data from Southwest China, our study showed that the rice dietary pattern has an inverse association with GD risks. Unexpectedly, aMED was positively associated with GD risks in our study. Findings were similar after additional adjustment for potential confounders and after exclusion of biliary sludges. This surprising finding seems to be contrary to previous research, as a typical MED dietary pattern has been extensively associated with numerous health benefits.^28^ Actually, research focusing on overall dietary patterns and risk of GD disease is limited. A cohort study of male American health professionals found that higher adherence to MED was associated with a lower risk of symptomatic GD disease.^10^ A similar result was found in a French cohort study.^11^ The MED diet pattern is typical in these studies, with dietary intake including vegetables, fruits, nuts, dairy, legumes, cereals, fish, meat and meat products, alcohol, MUFA/SFA, and olive oil. However, olive oil is rarely consumed in non-Mediterranean populations, resulting in a lack of high-quality sources of MUFA. Therefore, the high score of MUFA:SFA can only reflect a high consumption of vegetable oils in our study,^22^ as shown in eTable 4. The contrary effects of aMED in our study might be ascribed to the different types of oil intake. Furthermore, according to the single-food component analysis of aMED, we found that MUFA:SFA contributed most to the risk of GD, which might imply that a high-fat diet should be considered as a risk factor for GD (eTable 2).^29^

The rice dietary pattern, characterized by a poor and traditional agricultural dietary pattern, with higher consumption of animal oil, rice, preserved vegetables, alcohol, salt, soybean products, fresh vegetables, and tubers, was associated with decreased risk of GD in this study. Accordingly, most of the food components were plant-based. Previous research on the effects of a vegetarian diet (containing no meat or fish) remain contradictory.^17^^,^^30^^,^^31^ A British cohort study (*n* = 49,652) suggested that vegetarians had a moderately increased risk of GD compared with non-vegetarians.^16^ On the other hand, a recent study conducted in Taiwan (*n* = 6,002) found that a vegetarian diet was associated with a decreased risk of GD in women.^15^ Though the rice dietary pattern is not a vegetarian diet completely avoiding meat or fish, it also exerted a beneficial effect on the lower risk of developing GD. It is worth noting that animal oil was loaded higher in the rice dietary pattern, which is a source of SFA. Several studies showed that SFA might enhance the formation of cholesterol cholelithiasis.^32^ In fact, 60.3% of our study participants did not consume animal oil. In the highest score quintile of rice dietary pattern, 65.9% of participants consume oils blended with vegetable oil (including rapeseed oil, sesame oil, soybean oil, and peanut oil) and lard. Wang et al found that the oil mixture containing lard and soybean oil had a remarkable anti-obesity effect.^33^ Besides, other interactions between food or nutrient combinations are also possible. From the stratified analysis, the rice dietary pattern exerted stronger effects among females, older, urban, and obese participants, and those with diabetes, factors associated with higher rates of GD. A plant-based diet may help in the prevention of GD, especially in high-risk populations.

Interestingly, the coarse grain dietary pattern, also a plant-based diet, showed a positive association with GD prevalence. The lack of fresh vegetables might be a reason, since fresh vegetables were of benefit to reduce GD risk in the single-food component analysis (eTable 3). The modern dietary pattern was related to a lower risk of GD only when participants were under 60 years old and free of diabetes. The modern dietary pattern represents a shifted dietary pattern in China to some extent. More attention needs to be paid to nutrient balance, since China is experiencing a rapid diet shift from a plant-based diet as the economy continues to develop.

To our knowledge, the present study is the first large-scale study to investigate the association between dietary patterns and GD risks in China. The large sample size is the first strength of our study, which enabled adjustment for many potential confounders and stratified analysis with sufficient power. Besides, ultrasonography was used to determine the presence of GD instead of self-reported measures. Lastly, our study assessed the associations of both a priori and posteriori dietary patterns with GD risks, which provided complementary outcomes and a more comprehensive assessment of participants’ diet habits. However, limitations are also worth noting. First, the cross-sectional study was not able to determine a causal relationship between diet and GD. We tried to minimize the potential reverse causality by excluding self-reported cases. Second, it is hard to determine the types of GD (mostly classified as cholesterol and pigment GD) using ultrasonography, so the diet difference towards different types of GD cannot be evaluated. Third, the validity of oil and salt was not evaluated in our study. Although we assessed the reproducibility, the validity was hard to calculate, as it is not feasible to assess condiments intake in 24-hour dietary recall. Finally, although we adjusted for a variety of potential confounders, some possibility of residual or unmeasured confounding due to different lifestyles and various geographical conditions cannot be excluded.

### Conclusion

In conclusion, our findings suggest a protective role of the rice dietary pattern against the risk of GD. For high-risk populations, making some shift to a traditional agricultural diet might help with the primary prevention of GD. Still, these initial results need to be confirmed by future prospective studies.

## SUPPLEMENTARY MATERIAL

eFigure 1. Flow diagram of study participation. ^a^Total energy intake, macronutrients, and fatty acids were calculated using a modified food exchange list and representative food values from China Food Composition Tables. Unusual total energy intake was defined as <600 or >3,500 kcal/day for females and <800 or >4,200 kcal/day for males.

eTable 1. Scoring criteria for the aMED score in the CMEC study

eTable 2. Odds ratios of gallstone risks associated with each of the food components in aMED dietary pattern

eTable 3. Odds ratios of gallstone risks associated with each of the food components in FFQ

eFigure 2. Estimated associations by further excluding biliary sludge. Models adjusted for sex, age, ethnicity, urbanicity, marital status, highest education completed, annual family income, occupation, physical activity, smoking status, regular intake of beverage, regular intake of dietary supplements, total energy intake, body mass index, and history of diabetes. Dots and bars represent adjusted odds ratios and 95 % confidence intervals, respectively. The lowest quintile is the reference group. aMED, alternative Mediterranean diet.

eTable 4. Food distribution of food groups in quintiles of aMED dietary pattern score (Median [25th, 75th percentiles])

## References

[1] Kratzer W, Mason RA, Kächele V. Prevalence of gallstones in sonographic surveys worldwide. J Clin Ultrasound. 1999;27:1–7. 10.1002/(SICI)1097-0096(199901)27:1<1::AID-JCU1>3.0.CO;2-H 9888092

[2] Stokes CS, Krawczyk M, Lammert F. Gallstones: environment, lifestyle and genes. Dig Dis. 2011;29:191–201. 10.1159/000323885 21734384

[3] Shabanzadeh DM. Incidence of gallstone disease and complications. Curr Opin Gastroenterol. 2018;34:81–89. 10.1097/MOG.0000000000000418 29256915

[4] Wang F, Wang J, Li Y, . Gallstone disease and type 2 diabetes risk: a Mendelian randomization study. Hepatology. 2019;70:610–620. 10.1002/hep.30403 30515881

[5] Fairfield CJ, Wigmore SJ, Harrison EM. Gallstone disease and the risk of cardiovascular disease. Sci Rep. 2019;9:5830. 10.1038/s41598-019-42327-2 30967586 PMC6456597

[6] Goetze TO. Gallbladder carcinoma: prognostic factors and therapeutic options. World J Gastroenterol. 2015;21:12211–12217. 10.3748/wjg.v21.i43.12211 26604631 PMC4649107

[7] Stokes CS, Gluud LL, Casper M, Lammert F. Diets for primary prevention of gallbladder stones in adults. Cochrane Database Syst Rev. 2014;3. 10.1002/14651858.CD009918.pub2

[8] Mathur A, Megan M, Al-Azzawi HH, . High dietary carbohydrates decrease gallbladder volume and enhance cholesterol crystal formation. Surgery. 2007;141:654–659. 10.1016/j.surg.2006.11.008 17462466

[9] Tsai CJ, Leitzmann MF, Willett WC, Giovannucci EL. Long-term intake of trans-fatty acids and risk of gallstone disease in men. Arch Intern Med. 2005;165:1011–1015. 10.1001/archinte.165.9.1011 15883239

[10] Wirth J, Song M, Fung TT, . Diet-quality scores and the risk of symptomatic gallstone disease: a prospective cohort study of male US health professionals. Int J Epidemiol. 2018;47:1938–1946. 10.1093/ije/dyy210 30312404 PMC6280928

[11] Barré A, Gusto G, Cadeau C, Carbonnel F, Boutron-Ruault MC. Diet and risk of cholecystectomy: a prospective study based on the French E3N Cohort. Am J Gastroenterol. 2017;112:1448–1456. 10.1038/ajg.2017.216 28741614

[12] Tsai CJ, Leitzmann MF, Willett WC, Giovannucci EL. Dietary protein and the risk of cholecystectomy in a cohort of US women: the Nurses’ Health Study. Am J Epidemiol. 2004;160:11–18. 10.1093/aje/kwh170 15229112

[13] Nordenvall C, Oskarsson V, Wolk A. Fruit and vegetable consumption and risk of cholecystectomy: a prospective cohort study of women and men. Eur J Nutr. 2018;57:75–81. 10.1007/s00394-016-1298-6 27544676 PMC5847035

[14] Nordenvall C, Oskarsson V, Wolk A. Inverse association between coffee consumption and risk of cholecystectomy in women but not in men. Clin Gastroenterol Hepatol. 2015;13:1096–1102.e1. 10.1016/j.cgh.2014.09.029 25245628

[15] Chang CM, Chiu THT, Chang CC, Lin MN, Lin CL. Plant-based diet, cholesterol, and risk of gallstone disease: a prospective study. Nutrients. 2019;11:335. 10.3390/nu11020335 30720747 PMC6412457

[16] McConnell TJ, Appleby PN, Key TJ. Vegetarian diet as a risk factor for symptomatic gallstone disease. Eur J Clin Nutr. 2017;71:731–735. 10.1038/ejcn.2016.252 28272400

[17] Walcher T, Haenle MM, Mason RA, Koenig W, Imhof A, Kratzer W; EMIL Study Group. The effect of alcohol, tobacco and caffeine consumption and vegetarian diet on gallstone prevalence. Eur J Gastroenterol Hepatol. 2010;22:1345–1351. 10.1097/MEG.0b013e32833efdb2 20802339

[18] Wang J, Shen S, Wang B, . Serum lipid levels are the risk factors of gallbladder stones: a population-based study in China. Lipids Health Dis. 2020;19:50. 10.1186/s12944-019-1184-3 32192520 PMC7083041

[19] Chen CH, Huang MH, Yang JC, . Prevalence and risk factors of gallstone disease in an adult population of Taiwan: an epidemiological survey. J Gastroenterol Hepatol. 2006;21:1737–1743. 10.1111/j.1440-1746.2006.04381.x 16984599

[20] Su Z, Gong Y, Liang Z. Prevalence of gallstone in Mainland China: a meta-analysis of cross-sectional studies. Clin Res Hepatol Gastroenterol. 2020;44:e69–e71. 10.1016/j.clinre.2020.04.015 32446673

[21] Zhao X, Hong F, Yin J, ; China Multi-Ethnic Cohort (CMEC) collaborative group. Cohort Profile: the China Multi-Ethnic Cohort (CMEC) study. Int J Epidemiol. 2021;50:721–721l. 10.1093/ije/dyaa185 33232485 PMC8271196

[22] Xiao X, Qin Z, Lv X, . Dietary patterns and cardiometabolic risks in diverse less-developed ethnic minority regions: results from the China Multi-Ethnic Cohort (CMEC) Study. Lancet Reg Health West Pac. 2021;15:100252. 10.1016/j.lanwpc.2021.100252 34528018 PMC8383007

[23] Trichopoulou A, Bamia C, Trichopoulos D. Anatomy of health effects of Mediterranean diet: Greek EPIC prospective cohort study. BMJ. 2009;338:b2337. 10.1136/bmj.b2337 19549997 PMC3272659

[24] Chen LY, Qiao QH, Zhang SC, Chen YH, Chao GQ, Fang LZ. Metabolic syndrome and gallstone disease. World J Gastroenterol. 2012;18:4215–4220. 10.3748/wjg.v18.i31.4215 22919256 PMC3422804

[25] Stender S, Nordestgaard BG, Tybjaerg-Hansen A. Elevated body mass index as a causal risk factor for symptomatic gallstone disease: a Mendelian randomization study. Hepatology. 2013;58:2133–2141. 10.1002/hep.26563 23775818

[26] Yuan S, Gill D, Giovannucci EL, Larsson SC. Obesity, type 2 diabetes, lifestyle factors, and risk of gallstone disease: a Mendelian randomization investigation. Clin Gastroenterol Hepatol. 2022;20:e529–e537. 10.1016/j.cgh.2020.12.034 33418132

[27] Gu Q, Zhou G, Xu T. Risk factors for gallstone disease in Shanghai: an observational study. Medicine. 2020;99:e18754. 10.1097/MD.0000000000018754 32011459 PMC7220401

[28] Martini D. Health Benefits of Mediterranean Diet. Nutrients. 2019;11:1802. 10.3390/nu11081802 31387226 PMC6723598

[29] Del Pozo R, Mardones L, Villagrán M, . [Effect of a high-fat diet on cholesterol gallstone formation]. Rev Med Chil. 2017;145:1099–1105. 10.4067/s0034-98872017000901099 29424395

[30] Misciagna G, Centonze S, Leoci C, . Diet, physical activity, and gallstones—a population-based, case-control study in southern Italy. Am J Clin Nutr. 1999;69:120–126. 10.1093/ajcn/69.1.120 9925133

[31] Pixley F, Wilson D, McPherson K, Mann J. Effect of vegetarianism on development of gall stones in women. Br Med J (Clin Res Ed). 1985;291:11–12. 10.1136/bmj.291.6487.11 3926039 PMC1416200

[32] Tsai CJ, Leitzmann MF, Willett WC, Giovannucci EL. Long-chain saturated fatty acids consumption and risk of gallstone dis ease among men. Ann Surg. 2008;247:95–103. 10.1097/SLA.0b013e31815792c2 18156928

[33] Wang J, Yan S, Xiao H, . Anti-obesity effect of a traditional Chinese dietary habit-blending lard with vegetable oil while cooking. Sci Rep. 2017;7:14689. 10.1038/s41598-017-14704-2 29089626 PMC5665938

