# Supplementary material for: Dietary Patterns and Gallstone Risks in Chinese Adults: A Cross-sectional Analysis of the China Multi-Ethnic Cohort Study
Source: J Epidemiol. 2023 Sep 5;33(9):471–7. doi: 10.2188/jea.JE20220039 (PMC10409532; doi:10.2188/jea.JE20220039)
Supplement: Supplementary file 1 [file je-33-471-s001.pdf]

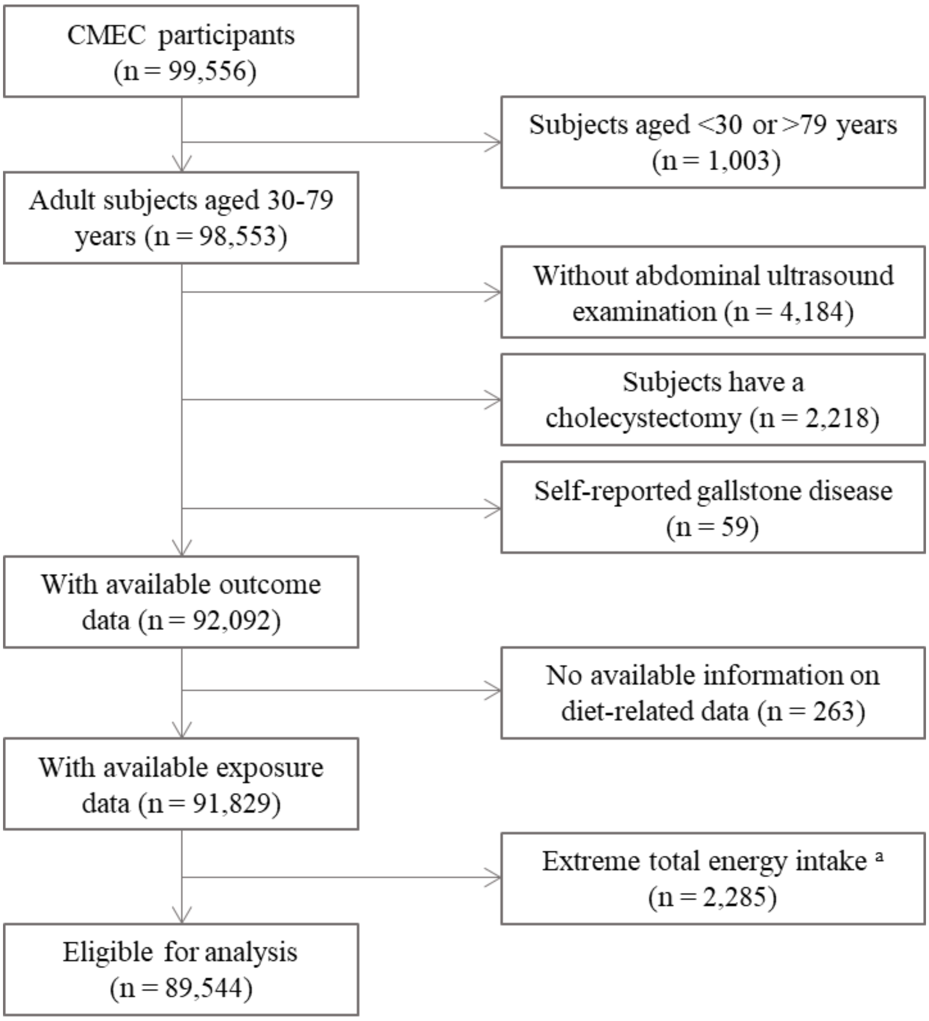

eFigure 1. Flow diagram of study participation. [a] Total energy intake, macronutrients, and fatty acids were calculated using a modified food exchange list and representative food values from China Food Composition Tables. Unusual total energy intake was defined as <600 or >3,500 kcal/day for females and <800 or >4,200 kcal/day for males.

eTable 1. Scoring criteria for the aMED score in the CMEC study

Component	Foods	Criteria for minimum score [a] (1)	Criteria for maximum score [a] (5)
Vegetables	All fresh vegetables except tubers and legumes	≤1,050.0 g/w	>3,150.0 g/w
Fruit	All fresh fruits	≤150.0 g/w	>1,400.0 g/w
Legumes	Soybeans, black beans, tofu, soybean milk, dried beans, dried bean curd	≤0.0 g/w	>100.0 g/w
Whole grains	Oats, sorghum, dried corn, highland barely	≤0.0 g/w	>225.0 g/w
Fish	Fish and all kinds of seafood products	≤0.0 g/w	>150.0 g/w
MUFA: SFA [b]	From all kinds of foods and fats	≤1.3 g/w	>1.9 g/w
Red and processed meats	Beef, mutton, pork, and their products	>1,050.0 g/w	≤150.0 g/w
Ethanol [c]	All alcoholic beverages	-	-

aMED, alternative Mediterranean diet; MUFA, monounsaturated fatty acids; SFA, saturated fatty acids.

[a] A score of 1 to 5 was assigned for each kind of food according to the quintile of the average food intake.

[b] Due to there being no values of fatty acids for food groups in the China food exchange list, we made an exchange value table according to the commonly consumed food items in each food group in Southwest China and the 2018 China food composition tables.

[c] According to the encouragement of moderate alcohol intake, the alcohol consumptions were categorized into five groups: (10,30], (0,10] or (30,40], 0 or (40,45], (45,50], and >50 grams per day for men; (5,15], (0,5] or (15,25], 0 or (25,30], (30,35], and >35 grams per day for women, and then we assigned descending scores of 1–5 to corresponding individuals.

eTable 2. Odds ratios of gallstone risks associated with each of the food components in aMED dietary pattern

Food components	OR[a] (95% CI)	*P* value
legumes	1.00 (0.98–1.02)	0.972
Fresh fruits	1.03 (1.01–1.05)	0.004
Fresh vegetables	0.98 (0.96–1.00)	0.084
Coarse grain	1.03 (1.01–1.05)	0.001
Red and processed meats	1.05 (1.02–1.08)	0.000
Fish/sea food	0.99 (0.97–1.01)	0.487
Alcohol	1.02 (0.98–1.06)	0.323
MUFA: SFA	1.06 (1.04–1.08)	0.000

aMED, alternative Mediterranean diet; CI, confidence interval; MUFA, monounsaturated fatty acids; OR, odds ratio; SFA, saturated fatty acids.

[a] Model adjusted for the potential confounders. Dietary components were scored as indicated in the construction of the aMED score, excepting that red and processed meats were fifths, ordered.

eTable 3. Odds ratios of gallstone risks associated with each of the food components in FFQ

Food components	OR[a] (95% CI)	*P* value
legumes	1.01 (0.99–1.03)	0.470
Fresh fruits	1.04 (1.01–1.06)	0.001
Fresh vegetables	0.98 (0.96–1.00)	0.044
Coarse grain	1.03 (1.01–1.05)	0.006
Red and processed meats	1.02 (0.99–1.04)	0.266
Fish/sea food	0.99 (0.97–1.01)	0.433
Alcohol	1.02 (0.98–1.07)	0.284
Tubers	1.01 (0.98–1.03)	0.592
Poultry	1.01 (0.99–1.03)	0.511
Eggs	0.87 (0.85–0.89)	0.000
Preserved vegetables	1.02 (1.00–1.03)	0.091
Rice	0.99 (0.96–1.02)	0.465
Wheat products	1.02 (1.00–1.04)	0.077
Vegetable oil	1.01 (0.99–1.04)	0.262
Tea	1.09 (1.03–1.16)	0.006
Dairy products	1.02 (0.97–1.08)	0.448
Animal oil	0.80 (0.75–0.86)	0.000

CI, confidence interval; FFQ, food frequency questionnaire; OR, odds ratio.

[a] Model adjusted for the potential confounders. Salt was excluded in the model because of the high collinearity with other food groups. Alcohol was scored as indicated in the construction of the alternative Mediterranean diet score. Tea, dairy products, and animal oil were dichotomized because of the granularity in the data (using medians as cut-offs). Other food groups were fifths, ordered.

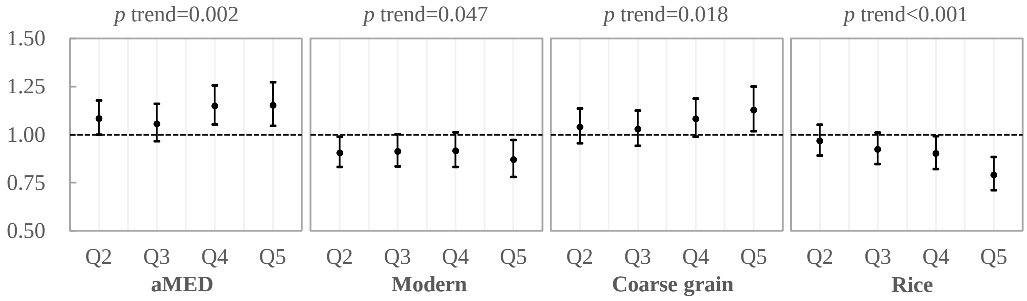

eFigure 2. Estimated associations by further excluding biliary sludge. Models adjusted for sex, age, ethnicity, urbanicity, marital status, highest education completed, annual family income, occupation, physical activity, smoking status, regular intake of beverage, regular intake of dietary supplements, total energy intake, body mass index, and history of diabetes. Dots and bars represent adjusted odds ratios and 95 % confidence intervals, respectively. The lowest quintile is the reference group. aMED, alternative Mediterranean diet.

eTable 4. Food distribution of food groups in quintiles of aMED dietary pattern score (Median [25th, 75th percentiles])

Food groups (g/w)	Q1	Q2	Q3	Q4	Q5
Alcohol	0.0 (0.0,5.0)	0.0 (0.0,5.0)	0.0 (0.0,5.0)	0.0 (0.0,5.0)	5.0 (0.0,5.0)
Tubers	50.0 (1.0,240.0)	100.0 (23.3,300.0)	140.0 (43.2,350.0)	160.0 (50.0,350.0)	200.0 (100.0,450.0)
Red and processed meats	700.0 (350.0,1400.0)	600.0 (210.0,875.0)	383.6 (200.0,700.0)	350.0 (175.0,700.0)	350.0 (150.0,700.0)
Poultry	23.3 (0.0,57.5)	35.0 (9.3,93.3)	46.7 (14.0,100.0)	50.0 (23.3,112.0)	70.0 (30.0,150.0)
Fish/sea food	1.9 (0.0,23.3)	23.3 (0.0,70.0)	46.7 (7.0,105.0)	70.0 (23.3,175.0)	144.7 (65.0,250.0)
Eggs	58.3 (1.9,200.0)	100.0 (23.3,240.0)	100.0 (35.0,300.0)	150.0 (50.0,350.0)	150.0 (58.3,350.0)
Fresh vegetables	1400.0 (700.0,2100.0)	1400.0 (1050.0,2100.0)	1750.0 (1400.0,2800.0)	2100.0 (1400.0,3150.0)	2800.0 (2100.0,3500.0)
Soybean products	0.0 (0.0,15.0)	14.0 (0.0,50.0)	30.0 (4.8,100.0)	46.7 (11.7,107.6)	70.0 (28.0,186.7)
Preserved vegetables	5.0 (0.0,50.0)	11.7 (0.0,50.0)	14.0 (0.0,50.0)	18.7 (0.4,60.0)	20.0 (1.9,70.0)
Fresh fruits	200.0 (46.7,460.0)	400.0 (150.0,908.0)	700.0 (300.0,1400.0)	1000.0 (450.0,1400.0)	1400.0 (700.0,1750.0)
Dairy products	0.0 (0.0,250.0)	11.5 (0.0,400.0)	58.3 (0.0,500.0)	116.7 (0.0,750.0)	240.0 (0.0,750.0)
Rice	2800.0 (1680.0,4200.0)	2800.0 (1683.5,4200.0)	2800.0 (2100.0,4200.0)	2800.0 (2100.0,4200.0)	2240.9 (1752.3,4200.0)
Wheat products	300.0 (46.7,700.0)	300.0 (93.3,700.0)	300.0 (100.0,700.0)	300.0 (105.0,700.0)	320.0 (150.0,700.0)
Coarse grain	0.0 (0.0,46.7)	11.5 (0.0,100.0)	35.0 (0.0,140.0)	70.0 (11.7,200.0)	143.0 (50.0,350.0)
Vegetable oil	172.6 (86.3,287.7)	230.1 (143.8,345.2)	265.5 (172.6,383.6)	287.7 (191.8,414.2)	345.2 (230.1,466.4)
Animal oil	0.0 (0.0,115.1)	0.0 (0.0,94.1)	0.0 (0.0,76.7)	0.0 (0.0,57.5)	0.0 (0.0,28.8)
Salt	40.3 (27.6,57.5)	40.3 (28.8,57.5)	40.3 (28.8,57.5)	39.8 (28.8,57.5)	40.3 (28.8,57.5)
Tea	0.0 (0.0,14.0)	0.0 (0.0,14.0)	0.0 (0.0,14.0)	0.0 (0.0,12.0)	0.0 (0.0,14.0)
